# Testing and Clinical Management of Health Care Personnel Potentially Exposed to Hepatitis C Virus — CDC Guidance, United States, 2020

**DOI:** 10.15585/mmwr.rr6906a1

**Published:** 2020-07-24

**Authors:** Anne C. Moorman, Marie A. de Perio, Ronald Goldschmidt, Carolyn Chu, David Kuhar, David K. Henderson, Susanna Naggie, Saleem Kamili, Philip R. Spradling, Stuart C. Gordon, Mark B. Russi, Eyasu H. Teshale

**Affiliations:** ^1^Division of Viral Hepatitis, National Center for HIV/AIDS, Viral Hepatitis, STD, and TB Prevention, CDC; ^2^Division of Field Studies and Engineering, National Institute for Occupational Safety and Health, CDC; ^3^National Clinician Consultation Center Post-Exposure Prophylaxis Hotline (PEPline), University of California at San Francisco Department of Family and Community Medicine, San Francisco, California; Zuckerberg San Francisco General Hospital, San Francisco, California; ^4^Division of Healthcare Quality Promotion, National Center for Emerging and Zoonotic Infectious Diseases, CDC; ^5^National Institutes of Health Clinical Center, Bethesda, Maryland; ^6^Duke Clinical Research Institute, Duke University School of Medicine, Durham, North Carolina; ^7^Division of Gastroenterology and Hepatology, Henry Ford Health System, Wayne State University School of Medicine, Detroit, Michigan; ^8^Occupational and Environmental Medicine Program, Yale University, Wellness and Employee Health, Yale-New Haven Health System, New Haven, Connecticut

## Abstract

Exposure to hepatitis viruses is a recognized occupational risk for health care personnel (HCP). This report establishes new CDC guidance that includes recommendations for a testing algorithm and clinical management for HCP with potential occupational exposure to hepatitis C virus (HCV). Baseline testing of the source patient and HCP should be performed as soon as possible (preferably within 48 hours) after the exposure. A source patient refers to any person receiving health care services whose blood or other potentially infectious material is the source of the HCP’s exposure. Two options are recommended for testing the source patient. The first option is to test the source patient with a nucleic acid test (NAT) for HCV RNA. This option is preferred, particularly if the source patient is known or suspected to have recent behaviors that increase risk for HCV acquisition (e.g., injection drug use within the previous 4 months) or if risk cannot be reliably assessed. The second option is to test the source patient for antibodies to hepatitis C virus (anti-HCV), then if positive, test for HCV RNA. For HCP, baseline testing for anti-HCV with reflex to a NAT for HCV RNA if positive should be conducted as soon as possible (preferably within 48 hours) after the exposure and may be simultaneous with source-patient testing. If follow-up testing is recommended based on the source patient’s status (e.g., HCV RNA positive or anti-HCV positive with unavailable HCV RNA or if the HCV infection status is unknown), HCP should be tested with a NAT for HCV RNA at 3–6 weeks postexposure. If HCV RNA is negative at 3–6 weeks postexposure, a final test for anti-HCV at 4–6 months postexposure is recommended. A source patient or HCP found to be positive for HCV RNA should be referred to care. Postexposure prophylaxis of hepatitis C is not recommended for HCP who have occupational exposure to blood and other body fluids. This guidance was developed based on expert opinion (*CDC. Updated U.S. Public Health Service guidelines for the management of occupational exposures to HBV, HCV, and HIV and recommendations for postexposure prophylaxis. MMWR Recommend Rep 2001;50[No. RR-11]; Supplementary Figure,* https://stacks.cdc.gov/view/cdc/90288) and reflects updated guidance from professional organizations that recommend treatment for acute HCV infection. Health care providers can use this guidance to update their procedures for postexposure testing and clinical management of HCP potentially exposed to hepatitis C virus.

## Introduction

Exposure to hepatitis viruses has long been recognized as an occupational risk for health care personnel (HCP), and recommendations previously were established for managing occupational exposures to bloodborne pathogens, including hepatitis C virus (HCV) (*1*) (Supplementary Figure, https://stacks.cdc.gov/view/cdc/90288). HCP might be exposed to blood or other body fluids, by injury from a used needle or from a splash of blood or body fluids into the eye or mouth, while caring for a patient. A source patient refers to any person receiving health care services whose blood or other potentially infectious material is the source of the HCP’s exposure. Although sharps injury prevention measures have led to overall exposure decreases in recent decades, blood and body fluid exposures, including sharps injuries, continue to occur (*2*). During 2018, a total of 34 U.S. hospitals reported through the Exposure Prevention Information Network (EPINet) a rate of 12.6 HCP blood and body fluid exposures per 100 average daily census days among the reporting hospitals (*3*). Similar exposures occur in other health care settings (e.g., nursing homes, clinics, and emergency departments) and during provision of in-home health care services.

## Background

For approximately 885 HCP with percutaneous exposure to anti-HCV–positive blood (72.7% of exposures) or body fluids (27.3% of exposures) during 2002–2015 in the United States, the estimated risk for HCV infection was reported as approximately 0.2% (two of 885; 95% confidence interval [CI]: 0%–0.52%) (*4*). HCV RNA status of the anti-HCV positive source patients was not described. Among 458 HCP with mucocutaneous exposure, the risk for HCV infection was 0% (95% CI: 0%–0.6%) (*4*). Recently published studies have reported similar infection risks with percutaneous exposure (*4*–*7*), although risks ranging from 0% to 10% have been reported from studies published earlier (*1*,*4*,*6*); variability might be explained in part by mechanism of injury, sensitivity of the test used to detect infection, and HCV RNA status of anti-HCV–positive source patients.

Transmission risk might be higher for HCP with exposure to hollow-bore needles (*4*,*8*). Challenge studies in chimpanzee animal models have demonstrated that an infectious titer (i.e., chimpanzee infectious dose) was required to transmit infection and that the needed inoculum was different in another animal model (i.e., human liver–chimeric mouse) (*9*). Data from one European case-control study of HCP who experienced seroconversion after exposure to an anti-HCV–positive source patient during 1991–2002 demonstrated that, among the limited number for whom source-patient HCV RNA status was known (n = 37; 62% of HCP who seroconverted), all source patients had been HCV RNA positive (*8*).

This report establishes new CDC guidance that includes recommendations for a testing algorithm and clinical management for HCP with potential occupational exposure to HCV, supplanting published recommendations (*1*) (Supplementary Figure, https://stacks.cdc.gov/view/cdc/90288). The new CDC guidance was developed on the basis of expert opinion and reflects current understanding of the viral dynamics of early HCV infection and recent guidance from the American Association for the Study of Liver Diseases (AASLD) and the Infectious Diseases Society of America (IDSA) that recommends treatment of acute HCV infection (https://www.hcvguidelines.org/unique-populations/acute-infection) (*10*). Testing guidance for source patients is described in consideration of the increasing incidence of acute HCV infection (*11*). Health care providers can use this guidance to update their procedures for postexposure testing and clinical management of HCP potentially exposed to hepatitis C virus.

## Methods

CDC developed this guidance with individual input and review by coauthors from federal agencies and academic and private health care institutions with subject matter expertise in occupational health and viral hepatitis epidemiology. The literature search described in the recent review of HCV incidence after HCP exposure through 2016 (*4*) was updated through October 2019, resulting in the inclusion of one additional reference (*5*). This guidance was presented to the Healthcare Infection Control Practices Advisory Committee for review and input at a public meeting in Atlanta, Georgia, on November 14, 2019. Subsequently, CDC made minor revisions to the figures for clarification to address the committee’s input.

## Rationale and Evidence

### Updated Guidance from Clinical Organizations

Recent guidance from AASLD and IDSA recommends a test-and-treat strategy for persons with acute HCV infection on initial diagnosis without awaiting spontaneous resolution (*10*). Although spontaneous clearance occurs in approximately 25% to 45% of acute infections (*1*,*12*,*13*), delays introduced by waiting for clearance might be associated with substantial anxiety on the part of the exposed HCP, might result in lost work time and risk for transmission depending on the HCP’s HCV RNA level (*14*), and might increase the possibility of loss to follow-up. Furthermore, emerging data about treatment of acute HCV infection with shortened courses of all-oral, direct-acting antiviral (DAA) regimens demonstrate potential benefit for treatment during the acute phase (*10*,*15*–*18*).

### Follow-Up Testing of HCV-Exposed HCP

For exposed HCP for whom follow-up testing is indicated, CDC continues to recommend early testing for HCV RNA at 3–6 weeks after exposure. HCV RNA becomes detectable on average within 1 week after exposure; most infected persons will have detectable HCV RNA within 1–2 weeks of exposure when tested with HCV RNA detection tests approved by the U.S. Food and Drug Administration (*19*,*20*). However, CDC now recommends additional follow-up testing at 4–6 months for anti-HCV with reflex or follow-up HCV RNA if anti-HCV positive because of the possibility of intermittent periods of aviremia during acute HCV infection. This phenomenon has been reported previously among exposed persons, including those who progressed to chronic infection, primarily when using older HCV RNA tests (*7*,*21*–*35*). Anti-HCV seroconversion occurs, on average, 8–11 weeks after exposure (*1*,*20*), although cases of delayed seroconversion have been documented among persons with immunosuppression (e.g., immunosuppression from human immunodeficiency virus [HIV] infection) (*36*,*37*). Frequency of testing during the follow-up period depends on the management objectives and plan for timing of therapy if seroconversion occurs.

### Increasing Incidence of Acute HCV Infection

In the United States, incidence of acute HCV infection is increasing, primarily related to injection drug use, with a 3.7-fold increase in cases reported to CDC during 2010–2017 (*11*). During 2014–2017, window-period infections (HCV RNA positive and anti-HCV negative) were identified among 5.3% of HCV RNA–positive deceased organ donors who had risk factors (*38*). These window-period data among a discrete population with recent behavioral risk for acquiring HCV indicate the possibility that, in certain health care settings, HCP might be exposed to source patients with early HCV infection before those patients develop serologic evidence of infection or symptoms indicative of viral hepatitis.

### Postexposure Prophylaxis

Postexposure prophylaxis (PEP) of hepatitis C is not recommended for HCP who have occupational exposure to blood and other body fluids (*1*,*10*,*39*–*41*) (Supplementary Figure, https://stacks.cdc.gov/view/cdc/90288). A 2017 publication estimated that 0.2% of percutaneous exposures result in HCV transmission (*4*). In contrast, older literature reported that approximately 1.8% of exposures resulted in transmission, meaning that routine PEP use for all occupational exposures would treat approximately 100 HCV-exposed persons for every two persons who might become infected (*40*). However, with the substantially lower 2017 transmission estimate of 0.2% for percutaneous exposures and 0% for mucocutaneous exposures (*4*), routine PEP would need to be administered to approximately 1,000 persons with percutaneous exposures for every two persons who might become infected, with no benefit to those with mucocutaneous exposures. Treatment efficacy and duration that would be required for HCV PEP has not been established (*40*,*41*). In 2019, a pilot trial of a 2-week DAA PEP regimen was initiated for HCP who were exposed from hollow-bore needlestick injury to an HCV RNA–positive source patient (*42*). Although the sample size will likely be insufficient for statistical power to determine whether PEP prevents seroconversion, this is the first DAA PEP study of HCV-exposed HCP. In contrast with other bloodborne pathogens for which PEP is recommended, curative DAA therapy is reserved for treatment if HCV transmission does occur (*10*,*15*–*17*,*40*,*41*).

## CDC Guidance and Recommendations

### Test the Source Patient

**FIGURE 1:**
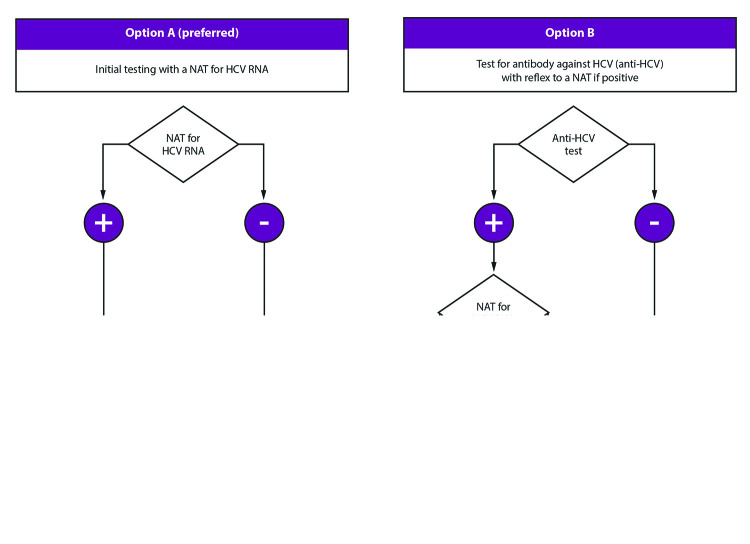
Testing of source patients after potential exposure of health care personnel to hepatitis C virus — CDC guidance, United States, 2020* **Abbreviations:** AASLD-IDSA = American Association for the Study of Liver Diseases and the Infectious Diseases Society of America; HCP = health care personnel; HCV = hepatitis C virus; NAT = nucleic acid test. * ***Testing of the source patient*** ***should be performed as soon as possible (preferably within 48 hour) after exposure.*** Testing may follow option A (preferred), which is testing with a NAT for HCV RNA, or option B, which is testing for anti-HCV with reflex to a NAT for HCV RNA if positive. If the source patient is known or suspected to have recent behaviors that increase risk for HCV acquisition (e.g., injection drug use within the previous 4 months) or if risk cannot be reliably assessed, initial testing of the source patient should include a NAT for HCV RNA. A source patient found to be positive for HCV RNA should be referred to care. Follow-up testing of HCP is recommended if the source patient is HCV RNA positive, anti-HCV positive with HCV RNA status unknown, or cannot be tested*.* ***Persons with detectable HCV RNA at any point*** should be referred to care consistent with current AASLD-IDSA guidelines for evaluation and treatment of all persons with acute or chronic HCV infection. Guidance for hepatitis C treatment (https://www.hcvguidelines.org) is evolving with emerging data on treatment with direct-acting antivirals.

Baseline testing of the source patient should be performed as soon as possible (preferably within 48 hours) after the exposure (*1*,*39*,*40*) (Figure 1) (Box). This guidance provides two options for initial source patient testing: 1) option A (preferred), to test for HCV RNA, or 2) option B, to test for anti-HCV and then if positive, test for HCV RNA (Figure 1). All source patients who are anti-HCV positive should be tested by a nucleic acid test (NAT) for HCV RNA (*43*), preferably with a reflex test by using the same specimen if cross-contamination is not a concern or by using a fresh aliquot of the same sample if stored correctly. If HCV RNA tests are positive but the RNA level is less than the lower limit of quantitation of the assay, the results are reported as <XX IU/mL (e.g., <15 IU/mL if the lower limit of quantitation of the assay is 15 IU/mL). This means that HCV RNA was detected in the sample but is not quantifiable and that the person from whom the sample was collected should be considered to have current HCV infection (*20*).

BOX **Testing of source patients and health care personnel potentially exposed to hepatitis C virus — CDC guidance, United States, 2020**

**Source-patient testing**

- Testing of the source patient may follow option A (preferred), which is testing with a nucleic acid test (NAT) for hepatitis C virus (HCV) RNA, **or** option B, which is testing for anti-HCV with reflex to a NAT if positive.
- If a source patient is known or suspected to have recent behaviors that increase risk for HCV acquisition (e.g., injection drug use within the previous 4 months) or if risk cannot be reliably assessed, initial testing should include a NAT.
- Follow-up testing of health care personnel (HCP) is recommended if the source patient is HCV RNA positive, anti-HCV positive with RNA status unknown, or cannot be tested.

HCP testing*

- Baseline testing of HCP for anti-HCV with reflex to a NAT if positive should be conducted as soon as possible (preferably within 48 hours) after the exposure and may be simultaneous with source-patient testing.
- If follow-up testing of HCP is recommended based on the source-patient’s status, test with a NAT at 3–6 weeks postexposure.
- If the HCP is NAT negative at 3–6 weeks postexposure, a final test for anti-HCV at 4–6 months postexposure is recommended.
- A source patient or HCP who is positive for HCV RNA should be referred to care.

* Follow-up testing of HCP is also warranted when concerns exist about specimen integrity, including handling and storage conditions that might have compromised source-patient test results, or if they exhibit any clinical signs of HCV infection.

If the source patient is known or suspected to have recent behavior risks for HCV acquisition (e.g., injection drug use within the previous 4 months) or if risk cannot be reliably assessed, initial testing should include a NAT for HCV RNA. Persons with recently acquired acute infection typically have detectable HCV RNA levels as early as 1–2 weeks after exposure (*19*,*20*). Source patients determined to be positive for anti-HCV or HCV RNA should be reported to the state or local health department (*11*) and referred for clinical management, as recommended (*10*). False-positive anti-HCV results are known to occur among populations at low risk (*44*).

HCV RNA testing is preferred for source patient testing. However, if anti-HCV testing is performed, a sufficient blood sample should be obtained for simultaneous or reflex (if anti-HCV positive) HCV RNA testing. This can minimize the need to redraw blood and reduce delays in establishing the status of the source patient. Testing of the source patient and baseline testing of the HCP might be either concurrent or sequential; follow-up testing of the HCP should be determined by the source patient’s status.

If the source patient is HCV RNA or anti-HCV positive with unavailable NAT or if the HCV infection status is unknown (e.g., when the HCP sustains a percutaneous injury from a needle in the trash), follow-up testing of the exposed HCP should be initiated. Follow-up testing for an HCP exposed to blood or body fluids from a source patient who tests anti-HCV positive but HCV RNA negative is not recommended because this status can indicate a previously cleared or cured infection. However, instances might occur when follow-up testing is warranted (e.g., when specimen integrity concerns exist, including handling and storage conditions, that might have compromised test results) (*20*) or if the HCP exhibits any clinical signs of HCV infection.

### Test the HCP

#### Baseline Testing

**FIGURE 2:**
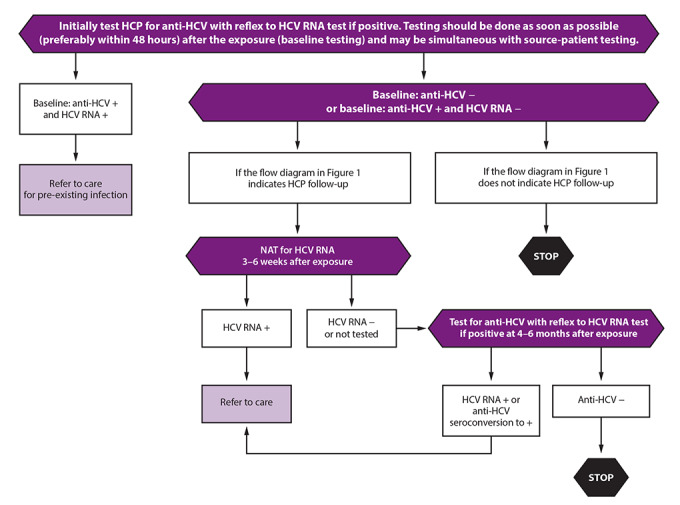
Testing of health care personnel after potential exposure to hepatitis C virus — CDC guidance, United States, 2020* **Abbreviations:** AASLD-IDSA = American Association for the Study of Liver Diseases and the Infectious Diseases Society of America; HCP = health care personnel; HCV = hepatitis C virus; NAT = nucleic acid test. * ***Baseline testing of HCP for anti-HCV with reflex to a NAT for HCV RNA if positive should be done as soon as possible*** (preferably within 48 hours) after the exposure and may be simultaneous with source-patient testing. ***If follow-up testing is recommended based on the source-patient’s status, test for HCV RNA at 3–6 weeks postexposure.*** Testing for HCV RNA performed at 6 weeks postexposure has the advantage of coinciding with human immunodeficiency virus (HIV) postexposure testing schedules if HIV surveillance is recommended. ***If HCV RNA is negative at 3–6 weeks postexposure, a final test for anti-HCV at 4–6 months postexposure is recommended*** due to the possibility of intermittent periods of aviremia in acute HCV infection. ***If the HCP was anti-HCV positive and HCV RNA negative at baseline, testing at this time should be conducted for HCV RNA detection, as persons successfully treated for HCV infection will remain anti-HCV positive and HCV RNA negative unless reinfected.*** Testing performed at 6 months postexposure has the advantage of coinciding with hepatitis B virus (HBV) postexposure testing schedules if HBV testing is recommended. ***HCP with anti-HCV seroconversion and negative HCV RNA should be referred for further evaluation.*** False-positive anti-HCV results are known to occur among low-risk populations. Anti-HCV seroconversion occurs on average 8–11 weeks after exposure, although cases of delayed seroconversion have been documented among persons with immunosuppression such as in HIV infection. ***For persons who had a negative anti-HCV result and are immunocompromised, testing for HCV RNA can be considered.*** Also, for persons with a positive anti-HCV and negative HCV RNA result, HCV RNA testing should be repeated if an additional potential HCV exposure occurred within the past 6 months, clinical evidence of HCV infection is present, or concerns exist about specimen integrity, including handling and storage conditions that might have compromised test results. ***Exposed persons who develop viral syndromes suggestive of acute HCV infection at any point*** should be retested for HCV RNA. ***Persons with detectable HCV RNA at any point*** should be referred to care consistent with current AASLD-IDSA guidelines for evaluation and treatment of all persons with acute or chronic HCV infection. Those persons with acute infection should be treated on initial diagnosis without awaiting spontaneous resolution. Guidance for hepatitis C treatment (https://www.hcvguidelines.org) is evolving with emerging data on treatment with direct-acting antivirals.

HCP should have an initial baseline test for anti-HCV with testing for HCV RNA if positive (i.e. either reflex or follow-up NAT) as soon as possible (preferably within 48 hours) after the exposure to rule out a pre-existing chronic infection (Box) (Figure 2). HCP testing positive for HCV RNA at baseline should be referred to care for pre-existing current HCV infection. If HCP are anti-HCV positive and HCV RNA negative at baseline, this likely indicates a previously cleared infection; therefore, if test results for the source patient warrant follow-up testing for HCP in context of a current exposure (Figure 1), HCP should be tested for HCV RNA instead of retesting for anti-HCV, which usually will remain positive regardless of current infection status.

#### HCV PEP Not Recommended

HCV PEP with DAA therapy is not routinely recommended. The risk for transmission of HCV from percutaneous exposures (0.2%) and mucocutaneous exposures (0%) is low (*4*) and in most situations does not justify giving DAAs to several hundred exposed HCP because of potential side effects; furthermore, efficient duration of PEP has not been established. DAA therapy is highly efficacious in eradicating acute and chronic infections (*10*,*15*–*17*,*40*,*41*); therefore, new HCV infections should be identified early and treated, and the strategy of testing and treating if transmission occurs is recommended.

#### Testing 3–6 Weeks Postexposure

If the source patient is HCV RNA positive or source-patient testing is not performed or not available, HCP baseline testing should be followed by a NAT for HCV RNA at 3–6 weeks after exposure. This test also should be performed if a source patient is anti-HCV positive and no source patient HCV RNA testing is available. A NAT performed at 6 weeks postexposure has the advantage of coinciding with HIV postexposure testing schedules, if recommended (*39*).

#### Testing 4–6 Months Postexposure

For all HCP for whom follow-up testing is recommended, a final test for anti-HCV at 4–6 months with testing for HCV RNA if positive (i.e. either reflex to or follow-up NAT) should be conducted (*1*,*10*,*39*,*40*). Testing performed at 6 months postexposure has the advantage of coinciding with hepatitis B virus (HBV) postexposure testing schedules, if recommended (*39*,*45*). Exposed HCP who develop illness with symptoms indicative of acute HCV infection at any point should be tested for HCV RNA.

No further follow-up is indicated for HCP who remain anti-HCV negative at 4–6 months. However, for those who had a negative anti-HCV result at 4–6 months and are immunocompromised or have liver disease, an additional test for HCV RNA can be considered (*20*). Seroconversion from anti-HCV negative to anti-HCV positive with undetectable HCV RNA can indicate resolved infection or acute infection during a period of aviremia (*31*). In addition, false-positive anti-HCV tests have been reported to occur (*44*). For HCP with a positive anti-HCV result and confirmed undetectable HCV RNA after 4–6 months, a NAT for HCV RNA should be repeated if clinical evidence of HCV infection is present (*31*). Tests should be repeated if concerns exist about results being compromised because of storage and handling errors or other issues that might affect specimen integrity (*20*).

#### Management of HCP Who Acquire HCV

HCP with detectable HCV RNA or anti-HCV seroconversion as a result of an occupational exposure should be referred for further care and evaluation for treatment as indicated in AASLD-IDSA guidelines (*10*). Because DAA therapy is highly efficacious in eradicating acute and chronic infections (*10*,*15*–*17*,*40*,*41*), new HCV infections should be identified early and treated (*10*). Additional recommendations are available to facilitate provision of occupational infection prevention and control services to HCP (*46*).
